# Food packaging-derived endocrine disruptors and breast cancer nutrition: dietary exposure, estrogen signaling, and survivorship implications

**DOI:** 10.3389/fpubh.2026.1909278

**Published:** 2026-08-05

**Authors:** Guang Yang, Xiang Meng

**Affiliations:** 1 Faculty of Science, Monash University, Melbourne, VIC, Australia; 2 Faculty of Science, University of Melbourne, Melbourne, VIC, Australia

**Keywords:** breast cancer, cancer nutrition, cancer survivorship, dietary exposure, endocrine disruptors, estrogen signaling, food contact chemicals, food packaging

## Abstract

Food packaging and other food contact materials are increasingly recognized as sources of dietary chemical exposure. Among the compounds that may migrate from packaging into foods, several classes are relevant to breast cancer nutrition because they can act as endocrine-disrupting chemicals, including bisphenols, phthalates, per- and polyfluoroalkyl substances, and selected non-intentionally added substances generated during food-contact material manufacture or use. These exposures do not replace established nutritional determinants of breast cancer outcomes, such as dietary quality, body weight, physical activity, and metabolic health. Rather, they represent an overlooked dimension of the food environment that may intersect with estrogen signaling, adipose inflammation, oxidative stress, epigenetic regulation, and treatment-related survivorship needs. Evidence linking packaging-derived endocrine disruptors to breast cancer remains heterogeneous and is strongest for mechanistic plausibility and exposure biology, while epidemiologic associations vary across compounds, timing of exposure, biomarkers, and tumor subtypes. This Mini Review synthesizes current evidence on food packaging-derived endocrine disruptors in relation to breast cancer nutrition, with emphasis on dietary exposure pathways, estrogen-related mechanisms, and practical survivorship implications. We propose that nutrition guidance for breast cancer survivors should include low-burden strategies to reduce avoidable food-contact chemical exposure, without promoting restrictive or anxiety-inducing dietary behavior. Future research should integrate dietary assessment, packaging-use patterns, biomonitoring, endocrine-metabolic biomarkers, and breast cancer outcomes to clarify modifiable exposure windows and prevention opportunities.

## 1 Introduction

Breast cancer remains the most commonly diagnosed malignancy among women worldwide and a major contributor to cancer-related morbidity and mortality (1). Advances in screening, surgery, systemic therapy, radiotherapy, and endocrine treatment have increased the number of women living with and beyond breast cancer, shifting clinical attention toward survivorship, metabolic health, recurrence prevention, treatment

tolerance, and quality of life. Nutrition is central to this agenda, and current survivorship guidance emphasizes healthy dietary patterns, weight management, physical activity, adequate protein intake, and limitation of excess alcohol and energy-dense foods (2–5).

Cancer nutrition guidance has traditionally focused on what patients eat. Yet diet is also shaped by how foods are processed, packaged, stored, heated, transported, and served. Food packaging and other food contact materials can release chemicals into foods, producing a form of dietary chemical exposure that is largely invisible to patients and clinicians (6–9). This matters for breast cancer because several food contact chemicals have endocrine-disrupting, metabolic, genotoxic, or mammary carcinogenesis-related properties, and environmental exposures are increasingly recognized as a component of breast cancer etiology (10–12).

This review does not claim that food packaging is a proven cause of breast cancer in individual patients; such a conclusion would exceed the current evidence. Instead, it frames packaging-derived endocrine-disrupting chemicals (EDCs) (13, 14) as a plausible, modifiable component of the nutritional exposure environment. The central question is how food contact chemical exposure may interact with breast cancer nutrition through estrogen signaling, adiposity, metabolic dysfunction, oxidative stress, and survivorship behavior. Framing food packaging in this way aligns it with cancer nutrition, because the exposure route is often dietary and because practical exposure-reduction strategies can be folded into survivorship nutrition counseling without compromising dietary adequacy.

## 2 Food packaging as a dietary exposure pathway

Food contact materials include plastics, paper and board, metal cans, coatings, adhesives, inks, rubber, silicone, glass, and multilayer materials used to store, package, process, prepare, or serve food. Chemicals may be intentionally added to confer flexibility, barrier function, heat resistance, grease resistance, color, adhesion, or durability. Other compounds arise as non-intentionally added substances, including impurities, degradation products, reaction by-products, or contaminants formed during manufacture, recycling, storage, or repeated use (7, 8).

Migration is influenced by both material properties and food conditions. Higher temperature, longer contact time, repeated use, high fat content, acidity, small package volume, and damaged or aged materials can increase migration for selected chemicals. Thus, a woman who frequently heats food in plastic containers, consumes canned foods with epoxy linings, stores fatty foods in flexible plastics, or relies heavily on packaged ultra-processed foods may experience a different exposure profile from another woman consuming similar nutrients from fresh or minimally packaged foods. This difference is important because traditional dietary assessment tools often capture food type but not packaging use, heating behavior, storage duration, or material contact.

**Figure 1:**
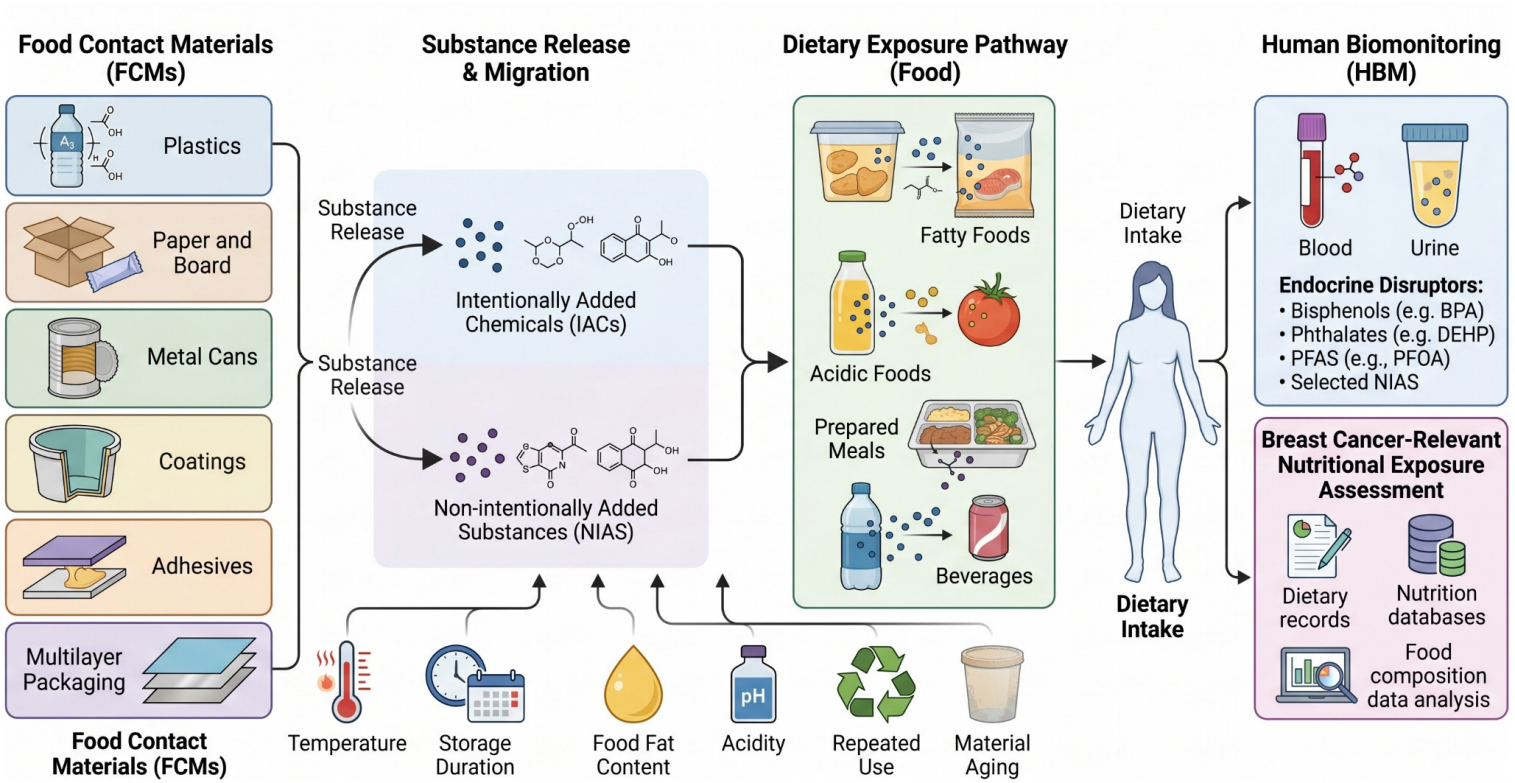
Food packaging as a dietary exposure pathway in breast cancer nutrition. Food contact materials, including plastics, paper and board, metal cans, coatings, adhesives, and multilayer packaging, can release intentionally added chemicals and non-intentionally added substances into food. Migration is modulated by temperature, storage duration, food fat content, acidity, repeated use, and material aging. Through these routes, packaging-derived endocrine disruptors such as bisphenols, phthalates, PFAS, and selected non-intentionally added substances enter the diet and become detectable in human biomonitoring, linking food packaging to breast cancer-relevant nutritional exposure assessment.

Large-scale evidence mapping has shown that many food contact chemicals are detected in humans. Geueke et al. (6) compared known food contact chemicals against biomonitoring and exposome resources and reported evidence of human exposure for 3,601 compounds, including substances with hazard properties of concern. Parkinson et al. (10) further identified potential mammary carcinogens detected in food contact materials, including chemicals found in plastics, paper and board, and other materials. These findings do not prove breast cancer causality, but they demonstrate that food packaging is a credible exposure source that should be considered in prevention-oriented nutrition research. The overall pathway from food contact materials to dietary exposure and human biomonitoring is illustrated in Figure 1.

Table 1 summarizes the major classes of food packaging-derived chemicals relevant to breast cancer nutrition, together with their principal packaging sources, dominant dietary exposure routes, key biological effects, and the current level of evidence for their relevance to breast cancer.

**Table 1:** Major food packaging-derived chemicals relevant to breast cancer nutrition: sources, exposure routes, biological effects, and evidence levels.

Chemical class	Main packaging sources	Primary dietary exposure routes	Key endocrine/biological effects	Evidence level for breast cancer relevance
Bisphenols (BPA, BPS, BPF)	Polycarbonate plastics; epoxy resin linings of cans and jar lids; thermal paper	Migration from can linings and polycarbonate containers, increased by heat, acidity, and prolonged storage	Estrogen receptor (ERα/ERβ) and GPER activation; altered aromatase activity, DNA methylation, and mammary cell proliferation	Strong mechanistic plausibility; inconsistent epidemiologic associations (15–20, 23, 24)
Phthalates (e.g., DEHP; metabolites MBzP, MiBP)	Flexible PVC films, gaskets, tubing, adhesives, and printing inks	Migration into fatty foods; contamination during food processing and handling	Anti-androgenic and adipogenic activity; insulin resistance, inflammation, and endocrine-metabolic disruption	Suggestive; some metabolites associated with risk in meta-analysis, others null (24, 26, 27)
Per- and polyfluoroalkyl substances (PFAS)	Grease-resistant paper and board, molded-fiber tableware, non-stick coatings	Migration from grease-resistant wrappers and coated packaging; also dietary and drinking-water sources	Persistence and bioaccumulation; immune, lipid, and endocrine effects; possible hormone-receptor heterogeneity	Weak/inconclusive overall; current epidemiologic evidence inadequate (28, 29)
Non-intentionally added substances (NIAS), oligomers, degradation products	Reaction by-products, residual monomers, ink components, recycled-material contaminants	Migration of chemically diverse, often unidentified mixtures	Largely uncharacterized; potential low-dose and mixture endocrine effects	Emerging/uncertain; limited data (7–9)
Microplastics and nanoplastics	Fragmentation of plastic packaging and containers	Ingestion of particles released into foods and beverages	Detected in human tissues and blood; possible carriers of adsorbed chemicals; biological role unclear	Preliminary; causal role in breast cancer unknown (30–32)

## 3 Packaging-derived endocrine disruptors relevant to breast cancer nutrition

### 3.1 Bisphenols

Bisphenol A (BPA) has long been used in polycarbonate plastics and in the epoxy resin linings of food and beverage cans, and its health effects have been reviewed extensively (15). Although BPA use has been restricted or re-evaluated in several jurisdictions (16), replacement bisphenols such as bisphenol S and bisphenol F remain a concern, since their structural similarity may preserve endocrine activity (17–19). Bisphenols can interact with estrogen receptor alpha, estrogen receptor beta, and the G protein-coupled estrogen receptor, activating downstream proliferative signaling in breast cell models (19, 20). Preclinical work further points to effects on aromatase, DNA methylation, mammary gland development, and endocrine therapy response (20–22).

Diet is a major route of bisphenol exposure, particularly through canned foods, plastic-wrapped products, and foods heated in certain plastics (17, 20, 23). The human evidence, however, is complex. Mechanistic studies offer plausible pathways, but epidemiologic studies of BPA and breast cancer risk have been inconsistent, likely reflecting exposure misclassification, the short half-life of BPA biomarkers, variation in the timing of exposure measurement, and heterogeneity across breast cancer subtypes (24, 25).

### 3.2 Phthalates

Phthalates are plasticizers used in flexible plastics and other consumer products. Although not all phthalate exposure derives from food packaging, diet can be an important route because these lipophilic compounds may migrate into fatty foods or enter the food chain through processing and handling (26). Several phthalates and metabolites have endocrine-disrupting, anti-androgenic, adipogenic, or metabolic effects (26, 27). A meta-analysis reported that some metabolites, including MBzP and MiBP, were associated with breast cancer risk, while BPA and several other metabolites were not consistently associated (24). These mixed findings support cautious interpretation rather than deterministic claims.

Phthalates may be particularly relevant in survivorship because they intersect with pathways already central to breast cancer nutrition: adiposity, insulin resistance, inflammation, and endocrine metabolism. Women with breast cancer are often advised to maintain healthy body weight and metabolic function. If packaging-related exposures contribute to endocrine-metabolic disruption, then reducing unnecessary exposure may be a complementary prevention strategy, although clinical outcome data remain insufficient.

### 3.3 PFAS and grease-resistant packaging

Per- and polyfluoroalkyl substances (PFAS) have been used in grease-resistant food packaging, non-stick coatings, and numerous industrial applications. PFAS are persistent, bioaccumulative, and associated with immune, lipid, endocrine, and carcinogenic outcomes in multiple evidence streams (28, 29). A recent systematic review and meta-analysis did not support a strong overall association between PFAS and breast cancer risk, but it observed possible heterogeneity by study design and hormone receptor status and highlighted the inadequacy of current epidemiologic evidence (28).

For breast cancer nutrition, PFAS illustrate a broader principle: exposure may not be determined solely by nutrient intake, but also by the food system and material environment. Packaged take-away foods, fast foods, grease-resistant paper, microwave popcorn bags, and some coated materials may contribute to exposure in certain settings. In survivorship counseling, the goal should not be to create fear of all packaged foods, but to support feasible substitutions when they also improve diet quality, such as choosing fresh or minimally packaged foods, using glass or stainless-steel storage, and avoiding unnecessary heating of food in disposable packaging.

### 3.4 Non-intentionally added substances, microplastics, and mixture exposure

Food contact materials can also contain non-intentionally added substances, including oligomers, degradation products, reaction by-products, residual monomers, contaminants, and chemicals from recycled materials (8). In addition, microplastics and nanoplastics can enter food and biological systems, although their causal role in breast cancer remains unknown (30–32). These exposures are difficult to study because they are chemically diverse, often co-occur as mixtures, and may not be captured by targeted biomonitoring.

Mixture exposure is especially relevant to breast cancer, because endocrine systems respond to low-dose, non-monotonic, and combined chemical effects. A patient is rarely exposed to a single compound in isolation. Packaging-derived chemicals typically co-occur with personal care product chemicals, household contaminants, air pollutants, diet-derived contaminants, and pharmaceutical exposures (33). This exposome-level complexity helps explain why mechanistic plausibility can be strong while epidemiologic attribution remains difficult.

## 4 Hormone-metabolic mechanisms linking food contact chemicals to breast cancer biology

Breast carcinogenesis and survivorship outcomes are shaped by estrogen and progesterone signaling, HER2 biology, immune contexture, adiposity, insulin resistance, inflammation, and genetic susceptibility. Packaging-derived EDCs may plausibly intersect with several of these pathways. The most direct route is estrogen receptor activation or modulation: bisphenols and certain other xenoestrogens can bind or activate estrogen-related signaling in breast cell models, promoting proliferation, migration, and gene-expression programs relevant to tumor biology (19, 20).

A related pathway is endocrine-metabolic remodeling. Obesity and post-diagnosis weight gain are associated with worse breast cancer outcomes in many studies, particularly among postmenopausal women (34). Adipose tissue drives aromatase-mediated estrogen production, inflammatory cytokine release, insulin resistance, and altered adipokine signaling, so EDCs that promote adipogenesis, disrupt PPAR signaling, or perturb lipid and glucose metabolism could compound the nutritional risk factors that matter for survivorship (25–27).

Genotoxicity and epigenetic disruption offer a further mechanism. A number of potential mammary carcinogens used in food contact materials are genotoxic or display key characteristics linked to mammary carcinogenesis (10, 11, 35). BPA and related compounds can alter DNA methylation and gene expression in breast epithelial models, affecting DNA repair, estrogen response, and mammary cell differentiation (21, 22). These effects are biologically important because the mammary gland is particularly sensitive to developmental windows, reproductive transitions, and endocrine perturbation.

**Figure 2:**
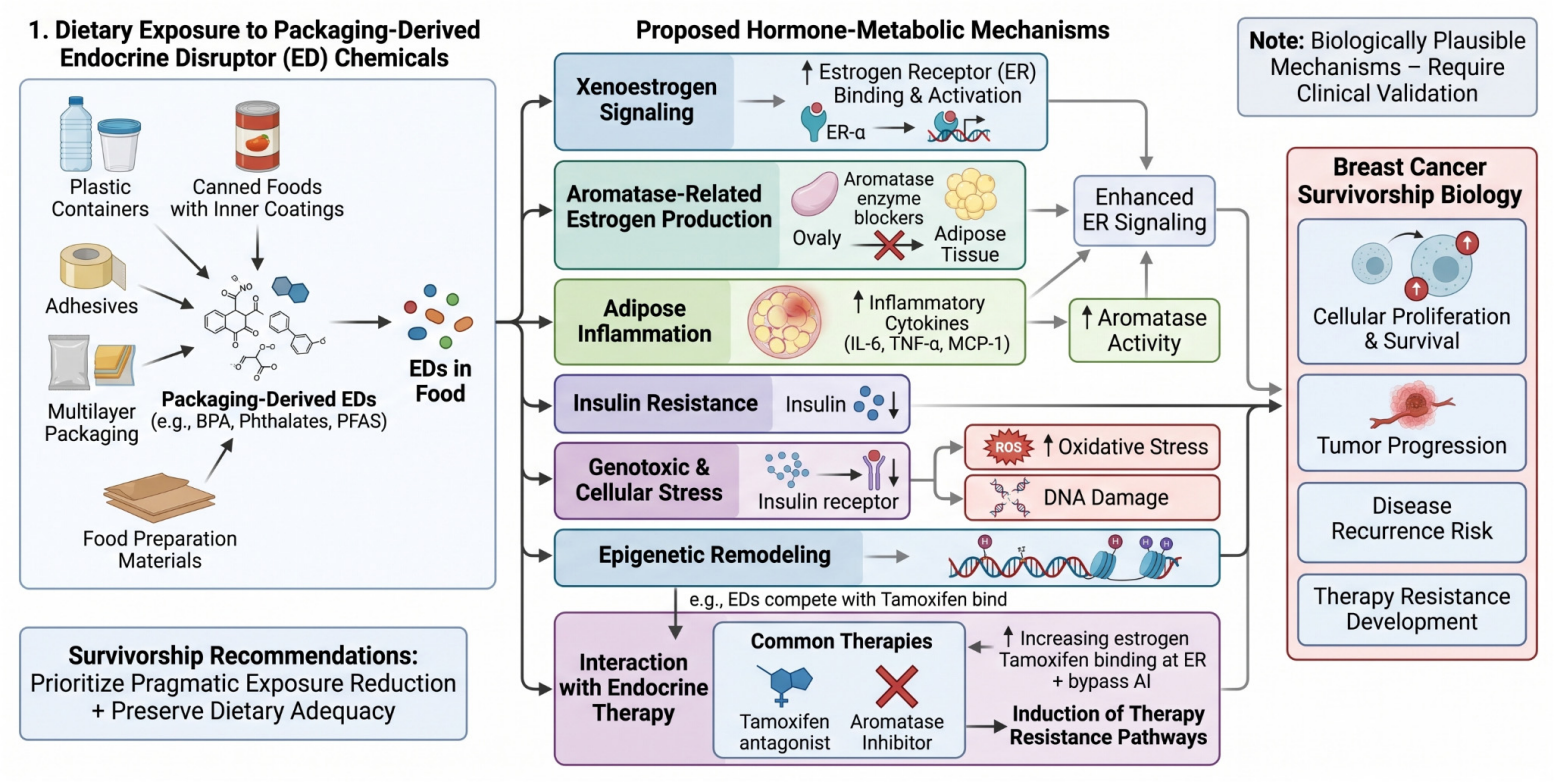
Proposed hormone-metabolic framework linking packaging-derived endocrine disruptors to breast cancer survivorship biology. Dietary exposure to food contact chemicals may influence estrogen receptor signaling, aromatase-related estrogen production, adipose inflammation, insulin resistance, oxidative stress, DNA damage, epigenetic remodeling, and endocrine therapy-related pathways. These mechanisms are biologically plausible but require clinical validation; accordingly, survivorship recommendations should prioritize pragmatic exposure reduction while preserving dietary adequacy.

Endocrine disruptors may also bear on treatment response. Laboratory studies indicate that some interact with anti-estrogen pathways, including tamoxifen-related signaling, in breast cancer cell models (20). Whether this translates to clinical effects is unknown, but it raises a legitimate question: could chronic low-level exposure to dietary EDC mixtures influence endocrine therapy response, metabolic side effects, or symptom burden? For now this remains a hypothesis in need of clinical validation rather than a basis for patient-level claims. The proposed hormone-metabolic mechanisms linking packaging-derived endocrine disruptors to breast cancer survivorship are summarized in Figure 2.

## 5 Epidemiologic evidence: suggestive, heterogeneous, and exposure-window dependent

Human evidence linking food packaging-derived EDCs to breast cancer is suggestive but heterogeneous. For bisphenols, preclinical data are stronger than epidemiologic evidence. Biomarkers of BPA exposure are limited by rapid metabolism and high within-person variability, making single urine measurements difficult to interpret (17, 24). For phthalates, some metabolites have shown associations with breast cancer risk in meta-analysis, while others have not (24). For PFAS, serum concentrations are more stable, but breast cancer findings remain inconsistent, with possible differences by timing of exposure assessment and hormone receptor status (28).

Several methodological factors explain this uncertainty. Breast cancer is heterogeneous, and estrogen receptor-positive, HER2-positive, and triple-negative tumors may differ in their susceptibility to endocrine and metabolic exposures. Timing also matters, since early-life, peripubertal, pregnancy, menopausal, and post-diagnosis exposure windows may carry different biological consequences. Packaging exposure is rarely measured directly; studies typically rely on urinary or serum biomarkers that integrate multiple sources, making it difficult to isolate food packaging from personal care products, dust, water, and occupational or environmental exposures. Diet quality and packaging exposure may also be confounded, because highly packaged foods are often ultra-processed, energy dense, and lower in fiber and micronutrients.

The most defensible conclusion, therefore, is not that food packaging-derived EDCs cause breast cancer, but that they represent a plausible and undermeasured dietary exposure pathway relevant to breast cancer prevention and survivorship. Advancing the field will require better exposure metrics, repeated biomarker measurements, tumor subtype-specific analyses, and closer integration with nutrition and metabolic data (36).

## 6 Survivorship implications for nutrition counseling

Breast cancer survivors already receive numerous dietary messages, many of which can be confusing or overly restrictive. Adding food packaging concerns must therefore be done carefully. Exposure-reduction guidance should be simple, low-cost, and compatible with nutritional adequacy. The goal is not to eliminate all packaged foods, which is unrealistic and may worsen anxiety or food insecurity, but to reduce avoidable high-contact exposures while supporting high-quality dietary patterns.

Practical strategies include avoiding the heating of food in plastic containers or disposable packaging, choosing glass, ceramic, or stainless-steel storage when feasible, reducing frequent consumption of canned foods when alternatives are available, limiting reliance on highly packaged ultra-processed foods, discarding damaged or aged plastic food containers, and avoiding repeated use of packaging not intended for reheating or long-term storage. These recommendations are broadly consistent with cancer survivorship nutrition more generally, since they tend to favor minimally processed foods, vegetables, fruits, legumes, whole grains, and home-prepared meals (2–4, 37).

However, equity must be considered. Some women rely on packaged foods because of cost, transport barriers, fatigue, work demands, disability, limited cooking facilities, or treatment-related symptoms. Counseling that assumes universal access to fresh foods or glass containers may be inappropriate. Clinicians should prioritize practical substitutions, such as not microwaving plastic, draining and rinsing canned foods when appropriate, selecting lower-packaging options within budget, and using community nutrition support when food access is limited. Exposure reduction should never replace evidence-based priorities such as treatment adherence, adequate energy and protein intake, management of treatment symptoms, physical activity, and psychosocial support.

## 7 Research priorities

Future work should bring together nutrition science, exposure assessment, oncology, and survivorship care. Dietary studies in breast cancer would benefit from capturing packaging-related behaviors, such as canned-food intake, heating food in plastic, take-away packaging, storage materials, and the frequency of packaged ultra-processed food consumption. Biomonitoring should move toward repeated measures and distinguish short-lived compounds, such as bisphenols and phthalates, from persistent chemicals, such as PFAS. Mechanistic studies, in turn, should test realistic mixtures at relevant exposure levels while accounting for estrogen receptor subtype, menopausal status, adiposity, and endocrine therapy.

Prospective cohorts are needed to link food contact chemical biomarkers with breast cancer subtype, recurrence, treatment response, metabolic outcomes, body composition, and quality of life. Intervention studies could then test whether feasible reductions in packaging exposure lower biomarker levels without compromising dietary adequacy or adding to patient burden. At the population level, policy research should evaluate whether safer food contact materials, improved chemical testing, better labeling, and substitution of hazardous compounds can reduce exposure without shifting costs onto consumers (38).

## 8 Conclusion

Food packaging-derived endocrine disruptors represent an overlooked dimension of breast cancer nutrition. The current evidence does not justify deterministic statements that food packaging causes breast cancer, but it supports a more nuanced view: food contact chemicals can migrate into foods, many are detectable in humans, and selected compounds have estrogenic, metabolic, genotoxic, or mammary carcinogenesis-related properties. For breast cancer prevention and survivorship, these exposures should be considered alongside established nutrition priorities, including healthy dietary patterns, weight management, physical activity, and metabolic health. Clinically, the most appropriate approach is pragmatic and equity-sensitive: reduce avoidable high-contact packaging exposures when feasible, avoid fear-based advice, and preserve dietary adequacy. Scientifically, the next step is to integrate packaging-use data, biomonitoring, endocrine-metabolic mechanisms, and breast cancer outcomes into prospective and intervention studies. Important evidence gaps nonetheless remain. Causal inference is constrained by short-lived biomarkers for several compounds, the limited direct measurement of packaging-specific exposure, and a scarcity of prospective and clinical data linking food contact chemical exposure to breast cancer incidence, recurrence, and treatment outcomes. Well-designed longitudinal cohorts with repeated biomonitoring, tumor subtype-specific analyses, and controlled clinical or intervention studies are therefore needed before firm conclusions or clinical recommendations can be drawn. Until such evidence is available, guidance should remain measured, acknowledging both the biological plausibility of these exposures and the current limitations of the human data.
